# The proteomic dataset for bone marrow derived human mesenchymal stromal cells: Effect of in vitro passaging

**DOI:** 10.1016/j.dib.2015.10.020

**Published:** 2015-11-01

**Authors:** Samuel T. Mindaye, Jessica Lo Surdo, Steven R. Bauer, Michail A. Alterman

**Affiliations:** a Tumor Vaccines and Biotechnology Branch, Division of Cellular and Gene Therapies, Center for Biologics Evaluation and Research, US Food and Drug Administration, Silver Spring, MD, United States; b Cellular and Tissue Therapies Branch, Division of Cellular and Gene Therapies, Center for Biologics Evaluation and Research, US Food and Drug Administration, Silver Spring, MD, United States

## Abstract

Bone-marrow derived mesenchymal stromal cells (BMSCs) have been in clinical trials for therapy. One major bottleneck in the advancement of BMSC-based products is the challenge associated with cell isolation, characterization, and ensuring cell fitness over the course of in vitro cell propagation steps. The data in this report is part of publications that explored the proteomic changes following in vitro passaging of BMSCs [4] and the molecular heterogeneity in cultures obtained from different human donors [5], [6].The methodological details involving cell manufacturing, proteome harvesting, protein identification and quantification as well as the bioinformatic analyses were described to ensure reproducibility of the results.

**Specifications Table**

**Table:** 

Subject area	*Biology, Chemistry*
More specific subject area	*Stem cell proteomics*
Type of data	*Protein and peptide identifications and quantification statistics followed by GO term enrichment using differentially regulated proteins*
How data was acquired	*The MS data was acquired using data-independent acquisition mode (UPLC–MS*^*E*^*(Synapt G2, Waters) and processed using PLGS, DAVID, PANTHER, Aray Track*
Data format	*CSV raw files exported from PLGS software and tables*
Experimental factors	*Bone-marrow derived mesenchymal stromal cells (BMSCs) were cultured and collected at three passage stages (P3, P5, and P7). Protein expression changes were comparatively analyzed using label-free proteomic technique*
Experimental features	*Total proteome was collected, proteins digested using trypsin, and known internal standards were spiked for relative quantification of proteins.*
Data source location	*US Food and Drug Administration, USA*
Data accessibility	*Data are included in this article,*S1 and S1_Exp

**Value of the data**

• BMSC cultures from five human donors (Table 1) were harvested at three different passages (P3, P5, and P7).
• The proteomic changes with in vitro aging of BMSCs were explored using label-free protein quantification technology.
• Across three passages, over 7000 proteins were identified and with the help of statistical filters differentially regulated proteins were identified.
• Extensive bioinformatic and GO enrichment analyses helped in describing the systematic changes in BMSCs induced by in vitro aging. Further, the data provide comprehensive proteomic architecture of BMSCs and thus serve as a rich source to identify molecular fingerprints of BMSCs.

## 1 Experimental design

### 1.1 Protein extraction using pressure cycling technology (PCT)

HBMSCs were thawed and dispersed in 50 mM Tris-HCl (Quality Biological, Gaithersburg, MD) containing 1% protease inhibitor cocktail (Sigma-Aldrich, St. Louis, MO), 20 mM dithiothreitol, DTT, (Pierce, Rockford, IL), and 1.25% sodium dodecyl sulfate (SDS). The suspension was sonicated in an ultrasonic cleaner (FS110D, Fisher Scientific, Pittsburgh, PA) for 60 min at 25 °C to ensure complete suspension of cells. The sample was transferred into a 1.4 mL single-use FT100-ND PCT pulse tube and placed in the Barocycler (NEP3229, Pressure Biosciences, South Easton, MA). Pressure in the tube was alternated for 20 cycles according to the following plan: high pressure ( 35,000 psi) for 20 s followed by ambient pressure for 10 s at room temperature. The temperature of the reaction chamber was controlled via a circulating water bath. The lysate was transferred to a LoBind tube (Eppendorf), spun at 16,100*g* for 10 min (model 5415D centrifuge, Eppendorf), and the supernatant was collected. The total protein content was determined using 2-D Quant kit (GE Healthcare, Piscataway, NJ) according to the manufacturer׳s instructions. The SDS was removed using Zeba spin desalting column (Thermo Scientific, Rockford, IL) as described in the user׳s guide, and protein solution was stored at −80 °C for further processing

### 1.2 GELFREE protein fractionation and digestion

The cell lysates from lines PCBM1632 and 167696 were fractionated into six using the gel-eluted liquid fraction entrapment electrophoresis (GELFREE) protein fractionation system (Protein Discovery, Knoxville, TN). Sufficient volume of cell lysate corresponding to 200 μg total protein was mixed with 30 μL acetate sample buffer, DTT to 50 mM final concentration, and the volume was adjusted to 150 μL using MS-grade water. The mixture was heated for 10 min at 50 °C and loaded onto an 8% tris-acetate cartridge (Protein Discovery, Knoxville, TN). Fractionation was carried out using the GELFREE 8100 protein fractionation system (Protein Discovery, Knoxville, TN). Prior to sample loading, HEPES buffer (Protein Discovery, Knoxville, TN) was filled into the anode and cathode reservoirs as well as the receiving chambers. Six fractions (F1–F6) were collected at 57.5, 61.5, 64.5, 68.5, 76.5, and 138.5 min from the time of loading using 50 eV for the first two fractions and 100 eV for the rest. Each time a fraction was collected; a fresh 150 μL HEPES buffer was introduced to collect the next round of fraction. From triplicate runs the respective fractions were combined and the volume was reduced to approximately 125 μL using SpeedVac Concentrator (ThermoFisher Scientific, Asheville, NC). Samples were then desalted using Pierce-detergent removal spin column (Thermo Scientific, Rockford, IL) and RapiGest SF (Waters Corp., Milford, MA) was added to 0.1% final concentration. Proteins were digested overnight using sequencing grade porcine trypsin (Promega, Madison, WI) following a standard procedure of reduction (10 mM DTT), alkylation (50 mM iodoacetamide), and quenching (10 mM DTT) prior to trypsin addition. The digestion was stopped by adding trifluoroacetic acid (TFA) to pH 2 and the RapiGest SF was hydrolyzed (37 °C for 30 min) and separated by centrifugation at 10,000*g* for 10 min. The digested samples were kept at −80 °C until further analysis. GELFREE fractionation step was omitted for cell lines from 110877, 8F3560, and PCBM1632. For these, tryptic digests were prepared directly from the whole cell lysates for reasons described in the paper.

### 1.3 MS data acquisition, processing, database searching, label-free quantification, and bioinformatic analyses

#### 1.3.1 LC–ESI–MS^E^ acquisition

2D RP/RP nanoLC separation of protein digests was performed using the nanoACQUITY UPLC system (Waters Corp.). The system was equipped with two binary solvent managers (BSMs), an autosampler, nano-tees, and switching valves. The first dimension BSM (1D BSM) eluted peptides at pH 10 from a fractionation column (XBridge C18, 300 μm, 5 mm, 3.5 μm, Waters Corp.), while the 2D BSM took eluent from 1D BSM, reduced the pH to 2 and decreased the organic content of the mobile phase through dilution to capture peptides on the trap column (Symmetry, C18, 300 μm, 5 mm, 5 μm, Waters Corp.). Peptides were then eluted using 2D BSM on the analytical RP column (Atlantis, 10 kpsi nanoAcquity, 75 μm, 100 mm, 1.7 μm, Waters Corp.).

For samples from cell lines 167696 and PCBM1632 (fractionated using GELFREE system as described in Section 1.2); 25 μL digest was aliquoted and volume-reduced to 10 μL using SpeedVac Concentrator, from which 2 μL (a full loop) was loaded onto the 1D RP column. A discontinuous gradient consisting of solvent A1 (20 mM ammonium formate, prepared from 28% NH_4_OH and FA solutions) and solvent B (0.1% formic acid (FA) in ACN) were used to elute peptides in six fractions (F1–F6) at 2 μL/min. Fractionation was done by eluting for 5 min according to the following gradient: F1 eluted at 11.1% B; F2 at 14.5%, F3 at 17.4%, F4 at 20.8%, F5 at 45.0%, and F6 at 65.0% B. The subsequent 2D separation involved a 60 min run at 0.3 μL/min using Solvent A2 (0.1% FA in H_2_O) and B. A linear gradient of 1–65% B in 30 min followed by an increase to 85% B in 1 min then decreased to 1% B in 5 min was used. A 24 min re-equilibration was introduced before the next fraction was eluted. The autosampler was maintained at 10 °C and the analytical column was kept at 35 °C, while the fractionation and trap columns remained at room temperature. The samples from cell lines 110877, 8F3560, and PCBM1662 were analyzed without prior GELFREE protein fractionation steps. Instead of protein fractionation, which significantly adds to the overall analysis time, the tryptic digest prepared from the total cell lysate was fractionated directly on the first RP column into 13 fractions prior to the second dimension separation on the analytical column. In this case a total of 7.5 µg digested sample was loaded on the the first RP column. The fractionation follows the following program: F1 eluted at 6% B; F2 at 7.5%, F3 at 9%, F4 at 11%, F5 at 12.6%, F6 at 14% B, F7 at 15.3%, F8 at 16.7% B; F9 at 18.3%, F10 at 20.4%, F11 at 23.5%, F12 at 50%, and F13 at 70% B. The subsequent 2D separation was performed as described for cell lines 2 and 3.

An online MS analysis was carried out using either SYNAPT-G2 (Waters Corp.). Acquisition using these instruments does not require the ion transmission window to be set during precursor ion scan prior to collision dissociation experiments. Rather, it alternates the collision energy between high and low levels (MS^E^) on a scan-by-scan basis to acquire all data points and achieves the maximum possible duty cycle [1]. The latter acquisition mode improves the sensitivity with subsequent improvement in the overall proteomic coverage because data are collected on all isotopes of every charge state across the entire chromatographic peak. The NanoAcquity UPLC and q-TOF MS were coupled through a nanoESI emitter (7 cm, 10 μm tip opening, New Objective). Both the UPLC and MS systems were controlled by Masslynx software, v.4.1 (Waters Corp.). A continuum positive ion was acquired via data-independent acquisition mode (DIA, MS^E^). During MS^E^ data acquisition, the quadrupole was set to transfer all ions between *m/z* 300 and 2000, while the collision cell alternate between low (4 eV) and high collision energies (15–45 eV) to record the abundances of precursor and fragment ions, respectively. To maintain the mass accuracy throughout the analyses, 2 ng/μL leucine enkephaline (Waters Corp.) was infused using a lock-spray apparatus and scanning was performed intermittently every 60 s for 3 s. Mass error corrections was performed post-acquisition during data processing. The TOF analyzer was externally calibrated using 100 fmol (Glu^1^)-fibrinopeptide (Waters Corp.) and all MS experiments were performed in v-mode with typical resolution of at least 10000 FWHM.

#### 1.3.2 Data processing and database searching

LC–MS^E^ raw data were processed using the Proteinlynx Global Server v.2.4 (PLGS) (Waters Corp.) [2]. The time alignment of MS^E^ data resulted in series of precursor and product ion tables. The product ion spectra were searched against Swiss-Prot human protein database using the PLGS. The search was limited to 10 ppm for the precursor and 20 ppm for fragment ion mass tolerances. Trypsin was set for the enzyme and up to 3 missed cleavages were allowed. Carbamidomethylation of cystein residues as fixed modification and oxidation of methionine and histidine residues for variable modifications were considered. In addition, the following limitation were set to facilitate the protein identification process: at least 3 product ion per peptide and 7 ion matches per protein, 100 ion counts for low and 75 ion counts for high energy acquisitions, and 1000 counts intensity threshold. The LC–MS^E^ data were also queried with one-time randomized version of the Swiss-Prot human protein database for the FDR calculation.

#### 1.3.3 Label-free protein quantification and bioinformatic analyses

**Fig. 1:**
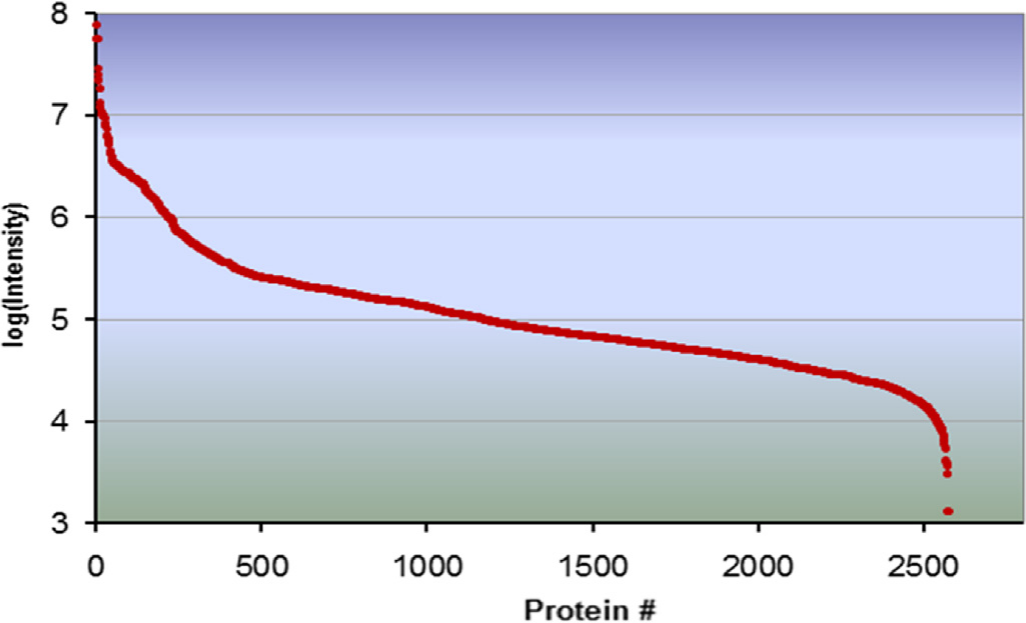
Dynamic range of proteins identified from hBMSCs (cell line PCBM1632). The average intensity of total product ions from triplicate runs was in log with base 10 plotted against protein accesion numbers.

**Fig. 2:**
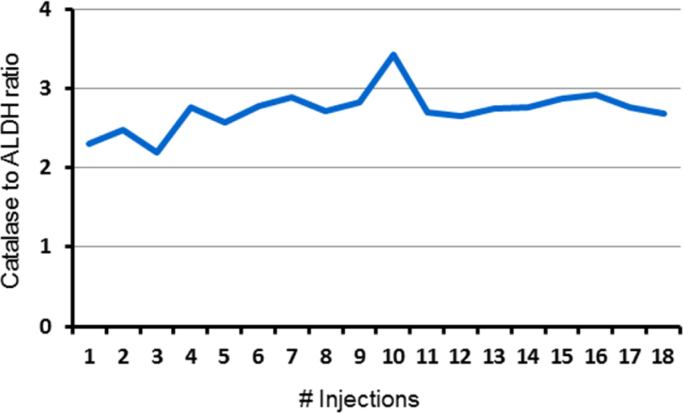
Performance evaluation of T3IP-based protein quantitation for hBMSC lysate. Catalase and ALDH (ratio 2.75:1) were mixed into MSC cell lysate and injected. I3P ratio was calculated after 2D fractionation and MS analysis.

**Fig. 3:**
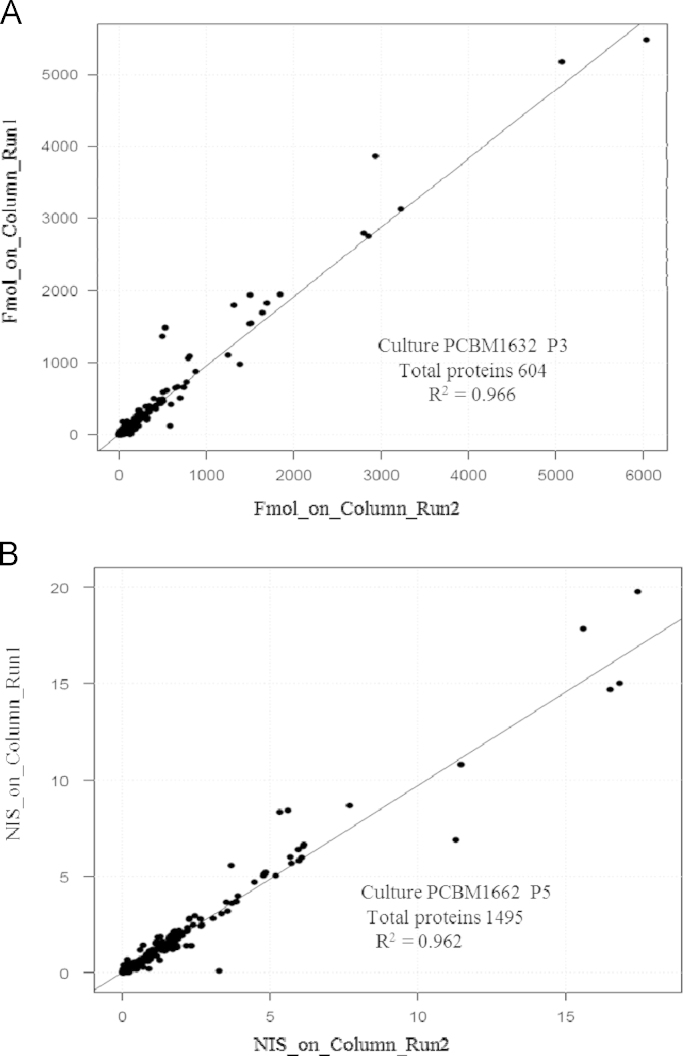
The run-to-run reproducibility of a label-free quantitation workflow. Protein abundance was determined using three most intense tryptic peptide signals. Panel A is an example, where offline protein fractionation step prior to trypsinization was used and B exemplifies a sample preparation workflow without prior protein fractionation.

Overall, over 700 proteins were confidently identified (See S1, S1_Exp and also [5], [6]) and as estimated by the average of protein-matched peptide intensity sum in triplicate runs at P3 the dynamic range of proteins expressed in hBMSCs spans 4–6 orders of magnitude (Fig. 1). The label-free protein quantitation method adopted in this study takes advantage of the linear relationship between MS signal response and protein concentration [8]. To assess the suitability of the protein quantification method for BMSC lysates, we spiked bovine catalase and alcohol dehydrogenase (ALDH) at the molar ratio of 2.75:1 to the hBMSC digest. Multiple runs of 2D nanoLC–MSE were acquired. The MS signal response ratios of catalase and ALDH over multiple injections in separate days was determined to be stable with an overall 16% coefficient of variation (Fig. 2). Furthermore, as applied to the actual hBMSCs proteome, the run-to-run quantitative repeatability was fairly robust as demonstrated in Fig. 3. Fig. 3A represents the run-to-run quantitative repeatability when digests were prepared with protein fractionation step (PCBM1632 at P3) prior to nanoLC–MSE analyses, while Fig. 3B represents a step without upfront protein fractionation (PCBM1662 at P5).

Subcellular location prediction for identified proteins was performed using PANTHER server [3] and GProX was used for clustering analysis [7]. Biological function, protein network assignment, and other bioinformatic analyses were performed using either the Ingenuity Pathway Analysis application (IPA v9, Ingenuity Systems Inc, USA) or ArrayTrack data analysis and interpretation tool (www.fda.gov/ScienceResearch/BioinformaticsTools/Arraytrack). In IPA, Core Analyses were run using the focus protein sets (differentially expressed proteins) together with the corresponding fold changes using the default set parameters. Networks and biological functions were algorithmically generated and significance of each network and enriched functions was assessed using Fisher׳s exact test. Furthermore, the activation z-score, which is a measure of regulation of biological functions, was calculated based on experimentally observed functional changes that are compiled in the Ingenuity Knowledge Database (IKDB).

## Figures and Tables

**Table 1:** Cell culture sources and donor characteristics.

Sample ID	Sex	Age	Source
PCBM1632	M	24	All cells
PCBM1662	F	31	All cells
167696	F	22	Lonza
110877	M	22	Lonza
8F3560	F	24	Lonza
